# A Cascaded Fabry–Pérot Interferometric Fiber Optic Force Sensor Utilizing the Vernier Effect

**DOI:** 10.3390/s25164887

**Published:** 2025-08-08

**Authors:** Zhuochen Wang, Ginu Rajan, Zhe Wang, Anuradha Rout, Yuliya Semenova

**Affiliations:** 1 Photonics Research Centre, School of Electrical and Electronic Engineering, Technological University Dublin, D07 ADY7 Dublin, Ireland; 2 Centre for Engineering Research in Intelligent Sensors and Systems, Cardiff School of Technologies, Cardiff Metropolitan University, Cardiff CF5 2YB, UK

**Keywords:** Fabry–Pérot fiber interferometer, Vernier effect, force sensor

## Abstract

An optical fiber force sensor based on the Vernier effect in cascaded Fabry–Perot interferometers (FPIs) formed by a barium tantalate microsphere and a section of polymethyl methacrylate (PMMA) optical fiber is proposed and investigated. Optical fiber sensors offer numerous advantages over their electronic counterparts, including immunity to electromagnetic interference and suitability for harsh environments. Despite these benefits, current optical fiber force sensors often face limitations in sensitivity, reliability, and fabrication costs. The proposed sensor has the potential to address these issues. Simulations and experimental results demonstrate that the sensor achieves a sensitivity of 9279.66 nm/N in a range of up to 3 mN. The sensor also exhibits excellent repeatability, making it a promising candidate for high-performance force monitoring in various challenging environments.

## 1. Introduction

The advantages of optical fiber sensors (OFSs) over their electronic counterparts are well known and widely recognized [1]. Their immunity to electromagnetic interference, electrical isolation, resistance to harsh environments, flexibility, multiplexing ability, and remote operation capability make OFSs a reliable choice for a broad range of applications such as measurements of temperature [2], PH level [3], humidity [4], force, and pressure [5]. Among them, optical fiber force sensors have been applied in fields ranging from industrial to biochemical, especially in challenging environments where high-performance force monitoring is crucial [6]. However, despite their many advantages, the high cost of equipment required to fabricate optical fiber sensors can be a drawback, limiting their practical applications.

Fabry–Pérot interferometric (FPI) optical fiber sensors are often used for force measurements due to their ultra-compact size, high sensitivity, and excellent thermal stability. More recently, cascading multiple FPIs to exploit the Vernier effect has been shown to improve the performance of FPI-based sensors [7]. The operating principle of FPI sensors is based on the dependence of the output transmission or reflection from the cavity on its length or refractive index (RI). Depending on the sensor’s design, both parameters may vary in response to an externally applied displacement, pressure, or force. In the open cavity configuration, the Fabry–Pérot cavity is directly in contact with the external environment, allowing for parameter detection by monitoring the shift in the reflection spectrum induced by changes in the cavity length or its RI [8].

Over the past few decades, FPI force sensors have been extensively researched. For example, in 2014, Chen et al. presented a compact, highly sensitive microfiber coupler-based reflective micro-force sensor [9]. The device was fabricated by fusing two twisted optical fibers and then connecting two pigtails to form a Sagnac loop with a high sensitivity of 3754 nm/N. In 2017, Liu et al. [10] proposed a strain and force sensor based on a fiber inline Fabry–Pérot (FP) microcavity, fabricated using a simple and cost-effective method that involved only fiber cleaving, tapering, and splicing, achieving a sensitivity of 841.59 nm/N. In 2018, Arnaldo et al. [11] proposed a force sensor based on polymer optical fiber Bragg gratings inscribed in cyclic, transparent, amorphous fluoropolymers, achieving a sensitivity of 34.17 nm/N. In 2024, Fu et al. [12] proposed a sensor based on two cascaded FP cavities that utilize the Vernier effect, achieving an ultra-high sensitivity of 1.40813 × 10^5^ nm/N. However, the above sensor required a relatively high-power laser source (800 mW).

Despite these advances, many existing sensors suffer from limited sensitivity, low reliability, high complexity, or high fabrication costs, making them unsuitable for mass production. This work proposes and investigates a new force-sensing structure composed of two cascaded fiber optic FPIs to utilize the Vernier effect. This proposed sensor offers high sensitivity and potentially lower fabrication costs, using a low-power optical source to address the limitations of current optical fiber force sensors.

## 2. Materials and Methods

AFPI is a well-established configuration extensively applied in lasers [13], telecommunications [14], and optical fiber sensing [15]. An FPI consists of two parallel reflecting surfaces separated by a certain distance, known as an etalon [16]. The interference within an FPI occurs due to multiple superpositions of reflected and transmitted beams within the resonator cavity. Constructive interference occurs when the beams are in phase, resulting in resonant enhancement within the resonator. Conversely, only a small portion of the launched light is stored inside the resonator when the beams are out of phase. Consequently, the stored, transmitted, and reflected light is spectrally modified compared to the incident light.

**Figure 1:**
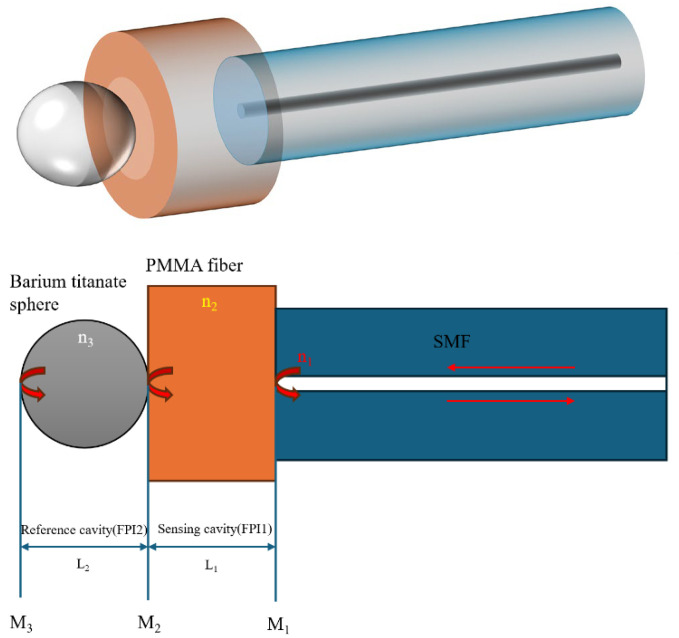
Schematic diagram of the proposed FPI sensor structure.

Figure 1 shows a schematic diagram of the proposed cascaded FPI sensor structure. The first interferometer is formed by a short section of a PMMA fiber, and an 80 μm diameter barium titanate microsphere forms the second cavity. Light from a broadband source launched into the SMF section experiences partial reflections at the interfaces between the two end faces of the PMMA fiber (M_1_ and M_2_), forming a sensing cavity.

A portion of light reflected at the interface between the PMMA fiber and the barium titanate microsphere is recoupled back into the SMF, where it interferes with the light reflected by the first PMMA interface. Meanwhile, a portion of the input light transmitted through the PMMA–microsphere interface undergoes further reflections at the opposite end of the sphere (M_3_), which, together with M_2_, forms the reference cavity.

The performance of FPI-based sensors can be characterized by several parameters. One of the critical parameters is the free spectral range (FSR) of the interference spectrum. The FSR is defined as the spectral distance between two adjacent interference wavelength peaks and is inversely proportional to the cavity length. A longer cavity results in a smaller FSR and vice versa. The expression for the FSR can be derived by first considering the interference conditions for the m-th and (m + 1)-th modes. The FSR of two cascaded interferometers (FPI1 and FPI2) can be calculated as follows [4]:

(1) $${FSR}_{1}=\frac{{\lambda_{0}}^{2}}{2n_{1}L_{1}}$$

(2) $${FSR}_{2}=\frac{{\lambda_{0}}^{2}}{2n_{2}L_{2}}$$

where *λ*_0_ is the central wavelength of the nearest transmission peak, *n* is the refractive index of the cavity medium, and *L* is its length. If the FSR values of the two cavities are comparable, the Vernier effect can be observed in the overall transmitted or reflected spectrum, with an FSR of [4]

(3) $$FSR=\frac{{FSR}_{1}*{FSR}_{2}}{\left|{FSR}_{1}-{FSR}_{2}\right|}$$

The enhancement of sensitivity can be illustrated by the magnification ratio M of the Vernier effect:

(4) $$M=\frac{FSR}{{FSR}_{1}}=\frac{{FSR}_{2}}{\left|{FSR}_{1}-{FSR}_{2}\right|}$$

The Vernier effect has been proven to be an effective method of enhancing the sensitivity of optical fiber sensors. The principle of the Vernier effect involves combining two interferometers with a slight difference in their FSRs and generating a superimposed spectrum, which allows for an increase in sensitivity by measuring the spectral shift of the “envelope.”

As mentioned above, the key requirement for achieving a “strong” Vernier effect is to ensure that the FSRs of the two cascaded interferometers are similar but not equal [7]. This can be achieved by adjusting the parameters of the two cascaded Fabry–Pérot cavities. In the proposed structure, light is reflected at multiple interfaces, including the end surfaces of the SMF, the PMMA fiber, and the opposite curved surfaces of the microsphere, which is placed in line with the SMF and PMMA fiber sections, leading to multimode interference. The PMMA fiber and barium titanate sphere form the cavities and mirrors. The use of PMMA ensures high force sensitivity due to its low Young’s modulus, while utilizing the barium titanate sphere enhances reflections and visibility of the interference spectrum due to its relatively high refractive index.

The reflection coefficients *R*_1_*, R*_2_, and *R*_3_ for each of the mirrors M_1_–M_3_, can be found as follows:

(5) $$R=\;{\left(\frac{n_{1}-n_{2}}{n_{1}+n_{2}}\right)}^{2}$$

where *n*_1_ and *n*_2_ are the refractive indices of the materials on both sides of the mirror surfaces.

The reflectance spectrum for the structure consisting of a PMMA cavity (FPI1) formed by M_1_ and M_2_ is a typical two-beam interference spectrum. The intensity of the light incident from the SMF returning to the SMF again after passing through the two reflecting surfaces can be expressed as follows [15]:

(6) $$I_{FPI1}=R_{1}+A^{2}+2R_{1}Acos(2\phi_{1})$$

where $A=(1-k_{1})(1-R_{1})\sqrt{R_{2}}$, and *k*_1_ is the transmission loss of the resonant cavity FPI1, *ϕ*_1_ is the phase shift due to light transmission in FPI1, *n*_1_ is the effective refractive index of PMMA, and *λ* is the wavelength of the input light in vacuum. Resonant troughs are found in the interference spectrum under the condition that the phase shift ϕ_1_ satisfies an even multiple of *π*. Due to the change in the interferometer cavity length, the phase shift changes with the applied force, which causes the wavelength at the center of the resonant trough in the interferometer spectrum to drift.

In the same way, the intensity of the interference spectrum of the incident light after passing through the entire cascaded structure (three reflectors) to form FPI2 can be expressed by

(7) $$I_{r}=R_{1}+A^{2}+B^{2}+2\sqrt{R_{1}}Bcos2\left(\phi_{1}+\phi_{2}\right)+2\sqrt{R_{1}}Acos\left(2\phi_{1}\right)+2ABcos\left(2\phi_{2}\right)$$

where $B=(1-k_{1})(1-k_{2})(1-R_{1})(1-R_{2})\sqrt{R_{3}}$, *k*_2_ is the transmission loss of the resonant cavity FPI2, *ϕ*_2_ is the phase shift due to light reflections in FPI2, and *n*_2_ is the effective refractive index of the barium titanate sphere.

The effect of a force applied to the structure in the axial direction can be analyzed by considering the contraction of the PMMA fiber as follows.

The force at the contact between the barium titanate microsphere and PMMA section can be calculated using explicit solutions for axially symmetric profiles following Johnson–Kendall–Roberts (JKR) theory [17]:

(8) $$F_{1}=\frac{4}{3}{E_{1}}^{*}{R_{1}}^{\frac{1}{2}}{d_{1}}^{\frac{3}{2}}$$

where *R*_1_ is the radius of the sphere, *d*_1_ is the combined decrease in the axial length of the sphere and PMMA fiber due to compression, and *E*_1_* is the effective Young’s modulus defined as follows:

(9) $$\frac{1}{E^{*}}=\frac{1-{v_{1}}^{2}}{E_{1}}+\frac{1-{v_{2}}^{2}}{E_{2}}$$

where *E*_1_ and *E*_2_ are the Young’s moduli, and *υ*_1_ and *υ*_2_ are the Poisson’s ratios associated with the barium titanate sphere and PMMA fiber. The Young’s modulus of PMMA is 3000 MPa [18], while the Young’s modulus of barium titanate is 150,000 MPa [19].

The force at the boundary between the PMMA fiber and SMF can be calculated as follows [17]:

(10) $$F_{2}=2R_{2}{E_{2}}^{*}d_{2}$$

where *R*_2_ is the radius of the SMF, *d*_2_ is the combined decrease in the axial length of the SMF and PMMA due to compression, and the *E*_2_* of the SMF and PMMA fiber can be calculated using Equation (9).

Due to the balance of forces, the axial forces on the sensor are equal:

(11) $$F=F_{1}=F_{2}$$

Since the Young’s moduli of barium titanate (microsphere) and silica (SMF) are much higher than the Young’s modulus of the PMMA, the compressed length of the sphere and SMF is much smaller than that of the PMMA fiber and can thus be neglected. Therefore, *d*_1_ and *d*_2_ can be seen as the compressed length of the PMMA at the sphere and the SMF sides, respectively. Assuming that the contractions in the axial lengths of both the barium titanate sphere and the SMF are small, the total decrease in the PMMA length is

(12) $$d=d_{1}+d_{2}$$

Therefore, the relationship between the force on the sensor and the decrease in the PMMA’s length *(d)* can be expressed as the following nonlinear function:

(13) d=3F4E1∗R123+F2R2E2∗

The relationship between the compressed length of the PMMA fiber and the applied axial force, as illustrated in Equation (13), is nonlinear. This suggests that the spectral shift resulting from compression exhibits a nonlinear correlation with the force. However, by restricting the force to a narrow range, the results can be regarded as approximately linear.

**Figure 2:**
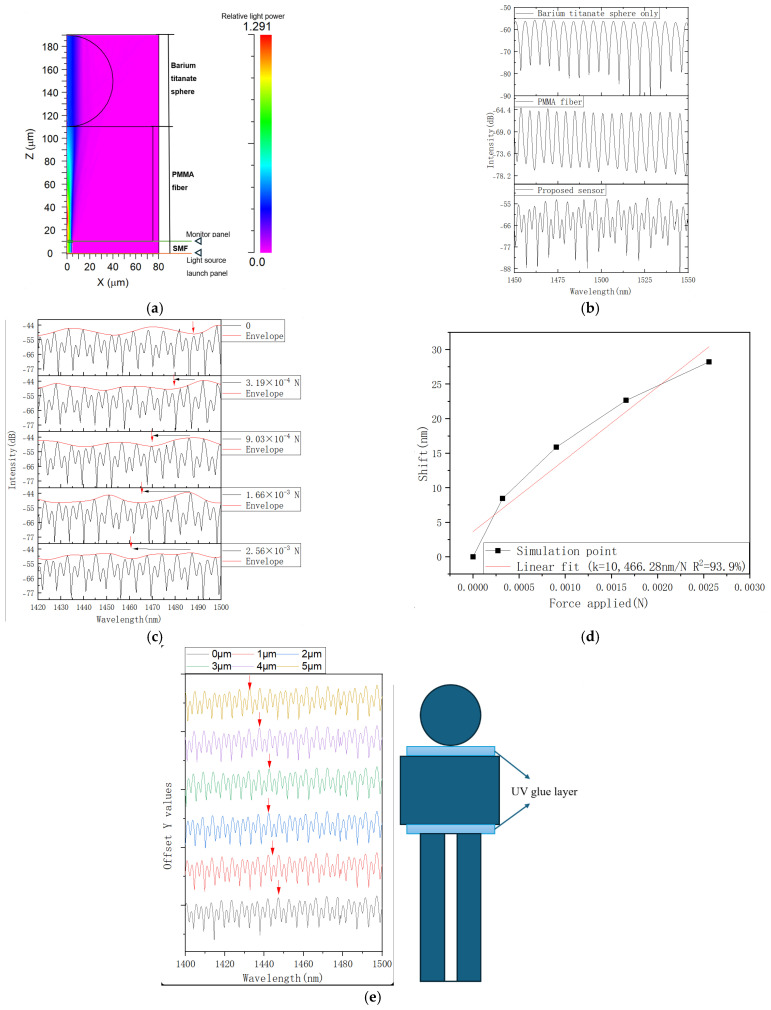
(**a**) Optical path model and optical field distribution at the free space wavelength of 1.5 µm; (**b**) simulated reflection spectra of the PMMA fiber cavity (top), barium titanate sphere cavity (middle), and cascaded PMMA–microsphere cavities; (**c**) simulated envelope spectra at different values of applied force; (**d**) spectral shift of the envelope versus applied force; (**e**) effect of UV glue layer and schematic of the simulation model.

To analyze the influence of the applied force on the reflection spectrum of the proposed structure in more detail, we conducted simulations using the RSoft software package (BeamPROP mode, Rsoft 2018). The optical path model diagram is shown in Figure 2a. In the simulation, the compressed length *d* of the PMMA fiber varied from 0 to 0.2 μm in 0.05 μm increments. The results of the simulations are presented in Figure 2b,d. We considered two interferometric cavities cascaded, as illustrated in Figure 1. The PMMA fiber and barium titanate cavities’ lengths were assumed to be 100 μm and 80 μm, and the corresponding refractive indices were set as 1.5 and 2.22. The surrounding refractive index was set to 1. X and Z coordinates in Figure 2a show the dimensions of the simulation area. The light source launch panel (SMF fiber mode, free space source wavelength of 1.5 μm) and the monitor panel are indicated in Figure 2a by arrows. The pathway monitor panel was set at the border between the PMMA fiber and the SMF with major backward mode and launch power type. The length of the SMF connecting the sensor to the light source was set to 1000 μm, and the SMF had core and cladding diameters of 8.3 μm and 125 μm, respectively. All materials’ properties were chosen from the RSoft database. The spectra in Figure 2b were simulated from 1.4 to 1.6 μm with 500 steps using the Beam PROP simulation tool.

The FSRs for the PMMA cavity, cascaded PMMA fiber–microsphere interferometers, and the Vernier envelope are estimated as 6.43 nm, 3.66 nm, and 27.6 nm, respectively. The simulated spectra of the Vernier envelope of the cascaded structure at different applied forces, ranging from 0 to 2.55 mN, are plotted in Figure 2c. The resulting sensitivity of the structure is estimated to be 10,466.28 nm/N from Figure 2d, which illustrates the spectral shift of the envelope as a function of the applied force. It should be noted that in Figure 2b,c, all spectra are separated along the vertical axis for better visibility, and arbitrary vertical offsets are introduced for the transmission values to illustrate more clearly how the spectrum shifts under the influence of the applied force, which shows a blue shift with an increase in force.

In this research, a PMMA-based UV glue was used to fix the sphere, PMMA fiber, and SMF with the layers’ thickness less than 2 μm. The simulation results related to the effect of the glue layers on the spectra of the proposed structure and the corresponding simulation model are illustrated in Figure 2e. The thicknesses of the glue layers were set to 0, 1, 2, 3, 4, and 5 μm. As one can see from the graphs, increasing the thickness of the glue layers leads to increased spectral shifts (up to 15 nm), changes in FSR (27 ± 2.8 nm), and power losses. However, for layers thinner than 2 μm, such effects are small and can be neglected. It should be noted that the variability in the UV glue layer thickness is likely to pose reproducibility issues. Thus, during fabrication, it is necessary to utilize automated dispensing and curing techniques to control the glue thickness within acceptable tolerances.

## 3. Sensor Fabrication

**Figure 3:**
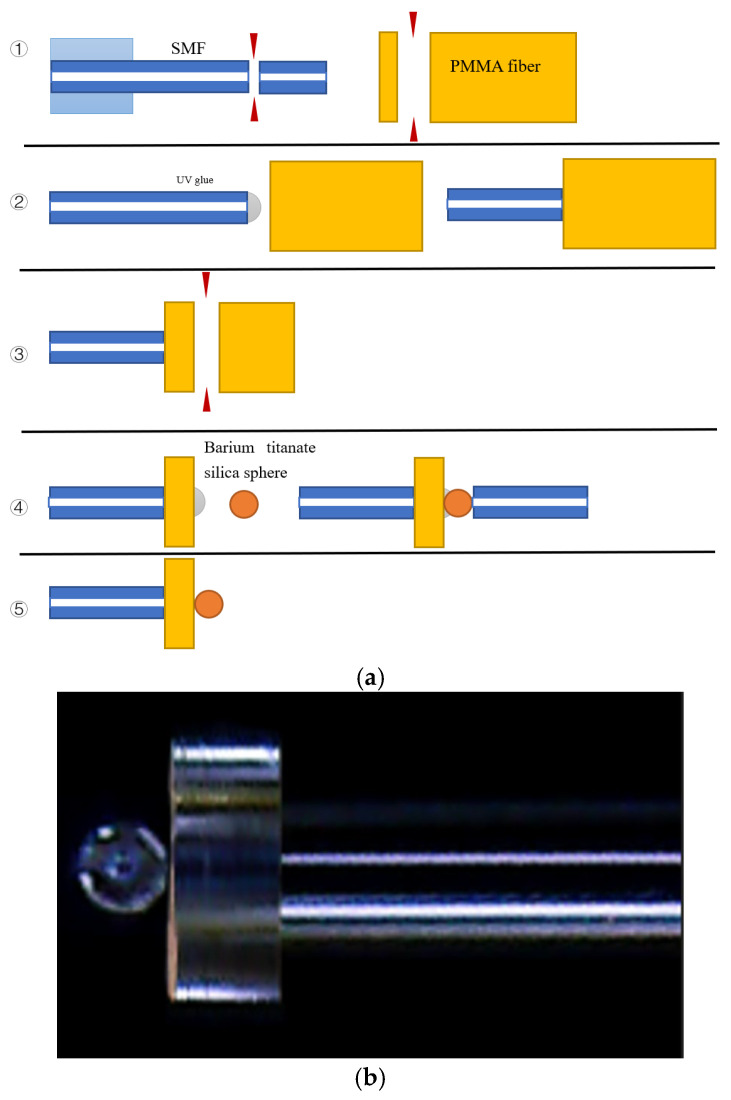
(**a**) Schematic diagram illustrating the fabrication process of the proposed structure and (**b**) microscopic image of the fabricated sensor.

The fabrication process for the proposed FPI structure is illustrated schematically in Figure 3a, which outlines a series of steps. Step 1: A short length of commercial single-mode fiber (SMF28) is used to launch the light from the optical source into the sensor via an optical circulator. Before connecting to the PMMA fiber section, the protective coating was stripped off from the end of the SMF using a stripper, and the fiber end was cleaved. Step 2: The cleaved SMF was then glued to the cleaved end of a short PMMA fiber (#57-096, Edmund Optics, core/cladding diameters 240/250 μm, *n_core_* = 1.492, *n_clad_* = 1.402, Barrington, NJ 08007, USA) section with a thin layer of UV-curable glue (AA350 Loctite, Berlin, Germany). A small force was applied along the fiber axis to ensure good physical contact before curing, which prevents UV glue from entering the gaps between the different parts and forming unwanted layers.

Step 3: After the UV glue dried, the free end of the PMMA was cleaved using a very sharp single-blade cutter, resulting in a length of approximately 103 µm. Step 4: A barium titanate sphere (BTGMS-ND2.2-4.38, Cospheric, *n_eff_* = 2.2, Somis, CA 93066, USA) was then glued onto the other cleaved end of the PMMA fiber section. Step 4: The end of the single-mode fiber was used to push all components (SMF, PMMA, and the barium titanate microsphere) until they came into physical contact. Step 5: The structure was left to dry for about 15 min. The entire fabrication process was conducted under a microscope to ensure the accurate axial alignment of all elements. The UV-curable glue (AA350 Loctite) is acrylate-based, like the PMMA, and therefore has similar optical properties. The thickness of the glue layer can be estimated from the microscopic image of the fabricated sensor shown in Figure 3b to be less than 2 µm, which was applied between the microsphere and PMMA fiber, and between the PMMA fiber and SMF.

## 4. Experimental Results and Discussion

**Figure 4:**
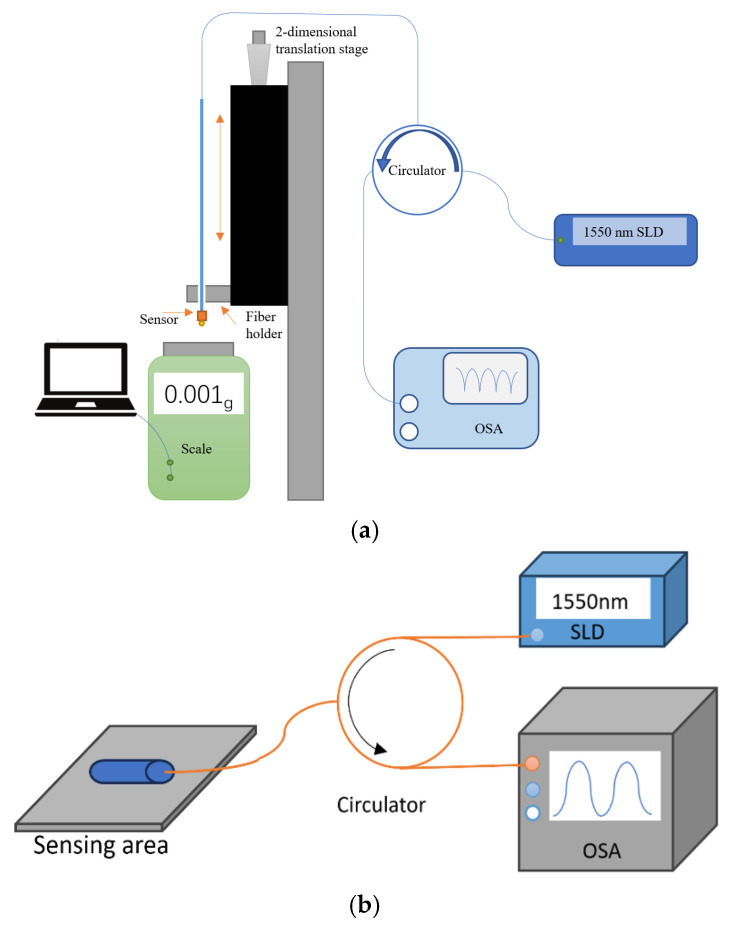
Schematic diagram of experimental setup: (**a**) sensing head and (**b**) overall optical characterization setup.

The schematic diagram of the experimental setup is shown in Figure 4. In this experiment, we utilized a broadband superluminescent light diode (SLED, 1550 nm, Thorlabs, Ely, UK) with a maximum power in the wavelength range of 1520–1580 nm. Light from the SLED was launched into the FPI through an optical circulator, and the reflected output spectrum was observed using a optical spectrum analyzer (OSA) with a 3 pm wavelength resolution. The structure of the proposed sensor has been described in Section 2.

The sensor was fixed on a two-dimensional translation stage using a fiber holder. The force was applied to the sensor by pushing it against the surface of a commercial electronic weight scale (Mettle Toledo, PL83-S, Greifense, Switzerland) vertically using the translation stage with a resolution of 0.5 µm. The weight scale had a resolution of 0.001 gf (gram-force), and its readings were sent to a PC through a 51MCU development board (STC89C52RC) and converted into Newtons (1 gf = 0.00980665 N). The first step was to move the sensor into physical contact with the scale, at which position the scale displayed 0.189 g (1.85 mN). Each subsequent step involved moving the sensor towards the scale by one micrometer, resulting in the corresponding readings of the scale: 0.314 g (3.08 mN), 0.477 g (4.68 mN), and 0.675 g (6.62 mN). All experiments followed the same steps to ensure a consistent test range. It should be noted that the SMF is essentially a connecting fiber, and its length has a negligible effect on the overall spectrum. Thus, the entire structure can be viewed as two cascaded FPIs.

**Figure 5:**
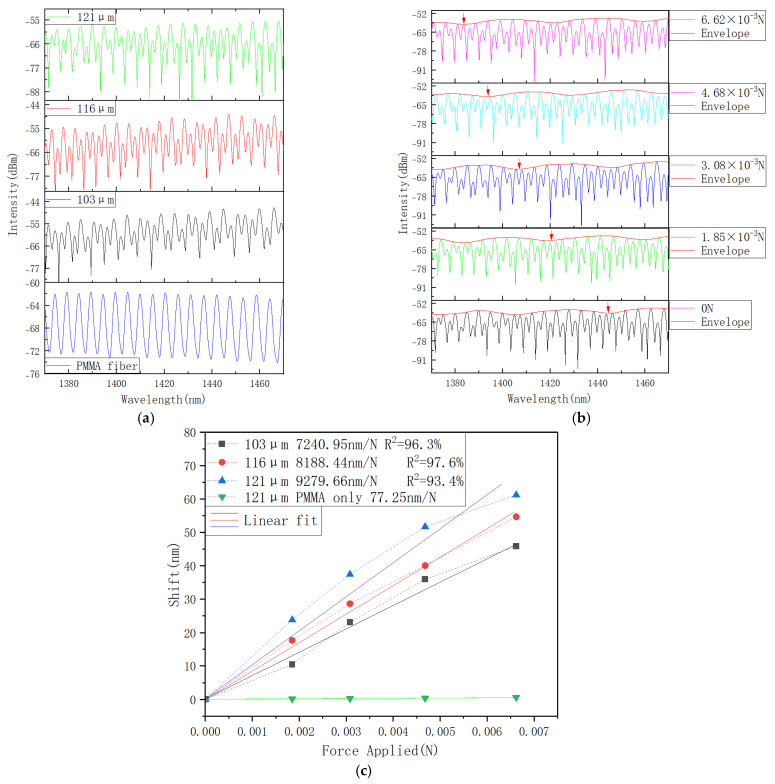
(**a**) Experimentally measured reflectance spectra of the sensor with different lengths of the PMMA fiber (121, 116, and 103 μm), and the spectrum for a 121-micron PMMA without the micro sphere (blue line); (**b**) measured reflection spectra of the structure with the PMMA length of 121 μm at different applied forces together with fitting envelopes; (**c**) corresponding spectral shifts versus applied force.

The experimental results are illustrated in Figure 5.

When the structure is compressed, the length of the PMMA cavity decreases, resulting in a blue spectral shift, as shown in Figure 5b. Similar to the graphs in Figure 2a,b, the data along the vertical axis in Figure 5b are plotted on the same graph for easier comparison only, and the transmission values are in arbitrary units.

As shown in Figure 5c, the sensitivity of the three samples with PMMA fiber lengths of 103, 116, and 121 μm is quite similar but shows a tendency to increase with the increase of the PMMA length, and the shift can be measured by tracking the peak shift of the envelope of each spectrum. They are calculated as 7240.55, 8188.44, and 9279.66 nm/N, corresponding to an approximate sensitivity improvement by a factor of 120 for all three samples compared to the result for the PMMA fiber without the microsphere, which is estimated at 77.25 nm/N. The experimental sensitivities agree with the simulated results, and the slight difference between the simulated and experimental results can be attributed to fabrication errors and the influence of the glue layers required to secure all parts of the sensor. The regression coefficients R^2^ of the three sensors are 96.3%, 97.6%, and 93.4%; therefore, within the proposed force sensing range, the results can be considered approximately linear. The results matched the relationship between the force and shift shown in the simulations. Compared to the simulation result, the experimental results showed lower sensitivity, which could be attributed to several reasons, for example, due to the influence of the glue on the Young’s moduli, propagation losses within the structure, and possible misalignment of the various contact surfaces within the structure, leading to a reduction in the axial force.

It should be noted that while the Vernier effect has been proven to improve sensitivity, the measurement accuracy of Vernier effect sensors may decrease compared with single-FPI sensors. Due to the Vernier effect, the resulting interference pattern of the proposed sensor, known as a Vernier fringe, has much wider spacing between the resonant peaks than those for the individual interferometers. This wider spacing is more sensitive to small changes. Small shifts in wavelength or phase that may be difficult to detect in a standard interferometer cause a much more significant change in the Vernier fringe. This magnification of the shift makes it easier to detect small changes, thus improving the sensor’s sensitivity. The measurement accuracy of a Vernier effect sensor depends on several factors, including the number of envelope-fitting data points, the signal-to-noise ratio at each data point, the full width at half maximum, and the valley contrast of the interference fringe [20]. To improve measurement accuracy in our experiments, we increased the number of measurement points to 1000 per scan and set the highest available wavelength resolution (0.03 nm) for the OSA. Furthermore, the measurement accuracy can be further increased by reducing the similarity of phase shifts between the two FPI cavities, resulting in a decrease in the FWHM of the fringes. However, this would lead to reduced sensitivity.

To mitigate this, in our future work, we aim to explore fine-tuning the difference between the FSRs of the cascaded FPIs to optimize the envelope’s full width at half maximum, thereby balancing fringe spacing and detectability. Implementing digital signal processing or machine learning-based fringe-tracking algorithms may also enhance accuracy without compromising sensitivity.

**Figure 6:**
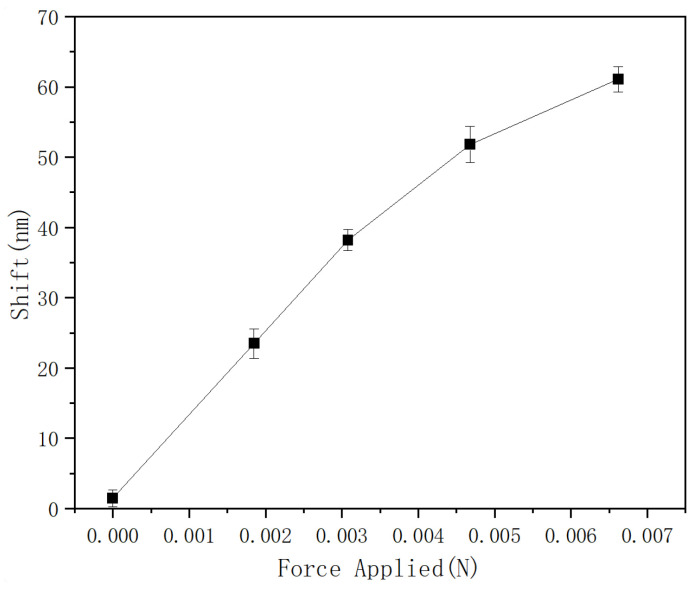
Repeatability tests for the proposed structure.

Finally, the repeatability of the proposed sensor’s performance was studied, and the corresponding results are presented in Figure 6.

The sensor with a 121 μm PMMA fiber length was used for repeatability testing. In this experiment, measurements were repeated five times within 48 h for the same sample, and the average value and error for every force-sensing point test are shown in Figure 6. The highest error, which occurred at a force value of 4.68 mN, is ±2.6 nm, and the smallest is ±1.2 nm. This means that, for the proposed sensor, the maximum sensing error is approximately ±0.3 mN, illustrating the sensor’s good repeatability. It is worth noting that the proposed sensor’s measurement range is relatively narrow. The FSR of the Vernier envelope primarily limits the measurement range, since an increase in the applied force beyond 3 mN would result in a spectral shift greater than the FSR of the envelope, leading to measurement ambiguity. It is possible to increase the FSR of the Vernier envelope by achieving a closer match between the FSRs of the cascaded interferometers, but this requires higher fabrication accuracy, and, on the other hand, decreases the accuracy in tracking of the envelope’s spectrum (as its spectral features become less sharp). Despite its relatively narrow range, the proposed sensor has numerous potential applications, including minimally invasive surgery, in robotics where it can be integrated into robotic grippers and hands, allowing robots to handle delicate objects without damaging them by providing feedback on the applied force, in assembly lines, where the proposed sensor can monitor the force exerted during the assembly process, and many others.

Table 1 compares the proposed structure with those previously reported in the literature.

As Table 1 shows, the proposed sensor offers competitive sensitivity compared with previously reported Vernier effect FPI force sensors. Moreover, compared to previously reported sensors, the proposed sensor offers an ultra-compact size (200 μm), a more straightforward fabrication process, and potentially lower cost. Compared to the sensor reported in [6], our sensor has lower sensitivity but can operate with a lower-power light source (22 mW versus 800 mW), resulting in reduced power consumption. The proposed sensor’s ultra-compact size and low power consumption make it ideal for integration into microsystem applications, such as minimally invasive biomedical instruments and confined robotic systems.

## 5. Conclusions

In conclusion, the proposed optical fiber force sensor structure, which combines a PMMA fiber and a barium titanate microsphere in a cascaded FPI configuration, exhibits high sensitivity and potentially lower fabrication costs compared to existing sensors. Using the Vernier effect significantly enhances the sensor’s performance, as demonstrated by both simulations and experimental results. The sensor achieves a sensitivity of 9279.66 nm/N over a force range of up to 3 mN with good repeatability. These attributes make the proposed sensor a viable, cost-effective solution for high-performance force monitoring in diverse industrial and biochemical applications. The proposed sensor offers advantages of low cost, high sensitivity, and compact size. Moreover, it does not require a high-power light source, thus reducing energy dependence. This sensor is particularly suitable for scenarios involving confined spaces and stringent power limitations, where real-time monitoring of slight force variations on devices or objects is essential. Future work will focus on further optimizing the sensor design and exploring its applications in real-world scenarios.

## Figures and Tables

**Table 1:** Comparison between the proposed sensor and previously reported sensors.

Reference	Sensitivity (nm/N)	Principle	Size (µm)	Source Power (mW)	Sensing Range (N)	Integration
[21]	26.1	FPI	<50	>20	0–3.92 × 10^5^	Easy
[6]	34.17	FBG	>500	>20	0–1.5 × 10^7^	Easy
[22]	17.527	Two-modal interferometer	8 × 10^4^	50	0–3 × 10^4^	Medium
[20]	4.4	SPR, POF	1 × 10^4^	4.7	0–5 × 10^5^	Medium
[12]	1.40813 × 10^5^	FPI-Vernier effect	250	800	0–1.4 × 10^3^	Difficult
This work	9.27966 × 10^3^	FPI-Vernier effect	200	22	0–3 × 10^−3^	Easy

## Data Availability

The original contributions presented in this study are included in the article. Further inquiries can be directed to the corresponding author.

## References

[1] Ferreira L.F., Antunes P., Domingues F., Silva P.A., André P.S. (2012). Monitoring of sea bed level changes in nearshore regions using fiber optic sensors. Measurement.

[2] Lee C.E., Taylor H.F. (1991). Fiber-optic Fabry-Perot temperature sensor using a low-coherence light source. J. Light. Technol..

[3] Zheng Y., Dong X., Ni K., Chan C.C., Shum P.P. Miniature pH sensor based on optical fiber Fabry-Perot interferometer. Proceedings of the 7th IEEE/International Conference on Advanced Infocomm Technology 2014.

[4] Liu Y., Gong H., Lu X., Ni K., Zhao C., Shen C. (2023). Optical fiber humidity sensor based on the Vernier effect of Fabry-Perot interferometers with a microsphere. Opt. Fiber Technol..

[5] Domingues M.F., Rodriguez C.A., Martins J., Tavares C., Marques C., Alberto N., André P., Antunes P. (2018). Cost-effective optical fiber pressure sensor based on intrinsic Fabry-Perot interferometric micro-cavities. Opt. Fiber Technol..

[6] Arata J., Terakawa S., Fujimoto H. (2013). Fiber Optic Force Sensor for Medical Applications within a Backbone-shape Structure. Procedia CIRP.

[7] Lin H.F., Liu F.F., Guo H.Y., Zhou A., Dai Y.T. (2018). Ultra-highly sensitive gas pressure sensor based on dual side-hole fiber interferometers with Vernier effect. Opt. Express.

[8] Chen H., Xie T., Feng J., Zhang X., Wang W., Li Y., Guo Z. (2022). A miniature fiber tip polystyrene microsphere temperature sensor with high sensitivity. Photonic Sens..

[9] Chen Y., Yan S.C., Zheng X., Xu F., Lu Y.Q. (2014). A miniature reflective micro-force sensor based on a microfiber coupler. Opt. Express.

[10] Liu Y., Lang C., Wei X., Qu S. (2017). Strain force sensor with ultra-high sensitivity based on fiber inline Fabry-Perot micro-cavity plugged by cantilever taper. Opt. Express.

[11] Li W., Liang T., Jia P., Lei C., Hong Y., Li Y., Yao Z., Liu W., Xiong J. (2019). Fiber-optic Fabry–Perot pressure sensor based on sapphire direct bonding for high-temperature applications. Appl. Opt..

[12] Fu H., Peng S., Li P., Teng C., Caucheteur C., Qu H., Hu X. (2024). Highly sensitive fiber force sensor based on cascaded Fabry-Perot cavities and Vernier effect. Opt. Laser Technol..

[13] Choi H.Y., Park K.S., Park S.J., Paek U., Lee B.H., Choi E.S. (2008). Miniature fiber-optic high temperature sensor based on a hybrid structured Fabry-Perot interferometer. Opt. Lett..

[14] Chen M., Zhao Y., Xia F., Peng Y., Tong R. (2018). High sensitivity temperature sensor based on fiber air-microbubble Fabry-Perot interferometer with PDMS-filled hollow-core fiber. Sens. Actuators.

[15] Qiu H.M., Jiang J.F., Yao L.L., Dai Z.P., Liu Z.Y., Qu H., Hu X.H. (2022). Ultrasensitive cascaded in-line Fabry-Perot refractometers based on a C-shaped fiber and the Vernier effect. Opt. Express.

[16] Zeng H., Geng T., An M. (2015). High temperature sensitivity fiber sensor based on M-Z interferometer fabricated by suspended dual-core hollow fiber. AOPC 2015: Optical Fiber Sensors and Applications.

[17] Popov V.L., Heß M., Willert E. (2019). Handbook of Contact Mechanics: Exact Solutions of Axisymmetric Contact Problems.

[18] Xu B., Liu Y., Wang D., Jia D., Jiang C. (2017). Optical fiber Fabry—Pérot interferometer based on an air cavity for gas pressure sensing. IEEE Photonics J..

[19] Liu Y., Wang Y., Yang D., Wu J., Zhang T., Yu D., Jia Z.N., Fu H. (2019). Hollow-core fiber-based all-fiber FPI sensor for simultaneous measurement of air pressure and temperature. IEEE Sens. J..

[20] Li Y.L., Li Y.H., Liu Y., Li Y., Qu S.L. (2022). Detection limit analysis of optical fiber sensors based on interferometers with the Vernier effect. Opt. Express.

[21] Liu Y., Qu S., Qu W., Que R. (2014). A Fabry—Perot cuboid cavity across the fibre for high-sensitivity strain force sensing. J. Opt..

[22] Zhao J., Jia D., Nie A., Zhang H., Liu T. (2020). Compact vectorial transverse force sensor based on two-modal interference in a few-mode seven-core fiber. J. Light. Technol..

